# The *Vibrio harveyi* master quorum-sensing regulator, LuxR, a TetR-type protein is both an activator and a repressor: DNA recognition and binding specificity at target promoters

**DOI:** 10.1111/j.1365-2958.2008.06389.x

**Published:** 2008-08-15

**Authors:** Audra J Pompeani, Joseph J Irgon, Michael F Berger, Martha L Bulyk, Ned S Wingreen, Bonnie L Bassler

**Affiliations:** 1 Department of Molecular Biology, Princeton UniversityPrinceton, NJ 08544, USA; 2 Division of Genetics, Department of Medicine, Brigham and Women's Hospital and Harvard Medical SchoolBoston, MA 02115, USA; 4 Department of Pathology, Brigham and Women's Hospital and Harvard Medical SchoolBoston, MA 02115, USA; 3 Committee on Higher Degrees in Biophysics, Harvard UniversityCambridge, MA 02138, USA; 5 Harvard/MIT Division of Health Sciences and Technology (HST); Harvard Medical SchoolBoston, MA 02115, USA; 6 Howard Hughes Medical InstituteChevy Chase, MD 20815, USA

## Abstract

Quorum sensing is the process of cell-to-cell communication by which bacteria communicate via secreted signal molecules called autoinducers. As cell population density increases, the accumulation of autoinducers leads to co-ordinated changes in gene expression across the bacterial community. The marine bacterium, *Vibrio harveyi*, uses three autoinducers to achieve intra-species, intra-genera and inter-species cell–cell communication. The detection of these autoinducers ultimately leads to the production of LuxR, the quorum-sensing master regulator that controls expression of the genes in the quorum-sensing regulon. LuxR is a member of the TetR protein superfamily; however, unlike other TetR repressors that typically repress their own gene expression and that of an adjacent operon, LuxR is capable of activating and repressing a large number of genes. Here, we used protein binding microarrays and a two-layered bioinformatics approach to show that LuxR binds a 21 bp consensus operator with dyad symmetry. *In vitro* and *in vivo* analyses of two promoters directly regulated by LuxR allowed us to identify those bases that are critical for LuxR binding. Together, the *in silico* and biochemical results enabled us to scan the genome and identify novel targets of LuxR in *V. harveyi* and thus expand the understanding of the quorum-sensing regulon.

## Introduction

**Fig. 1:**
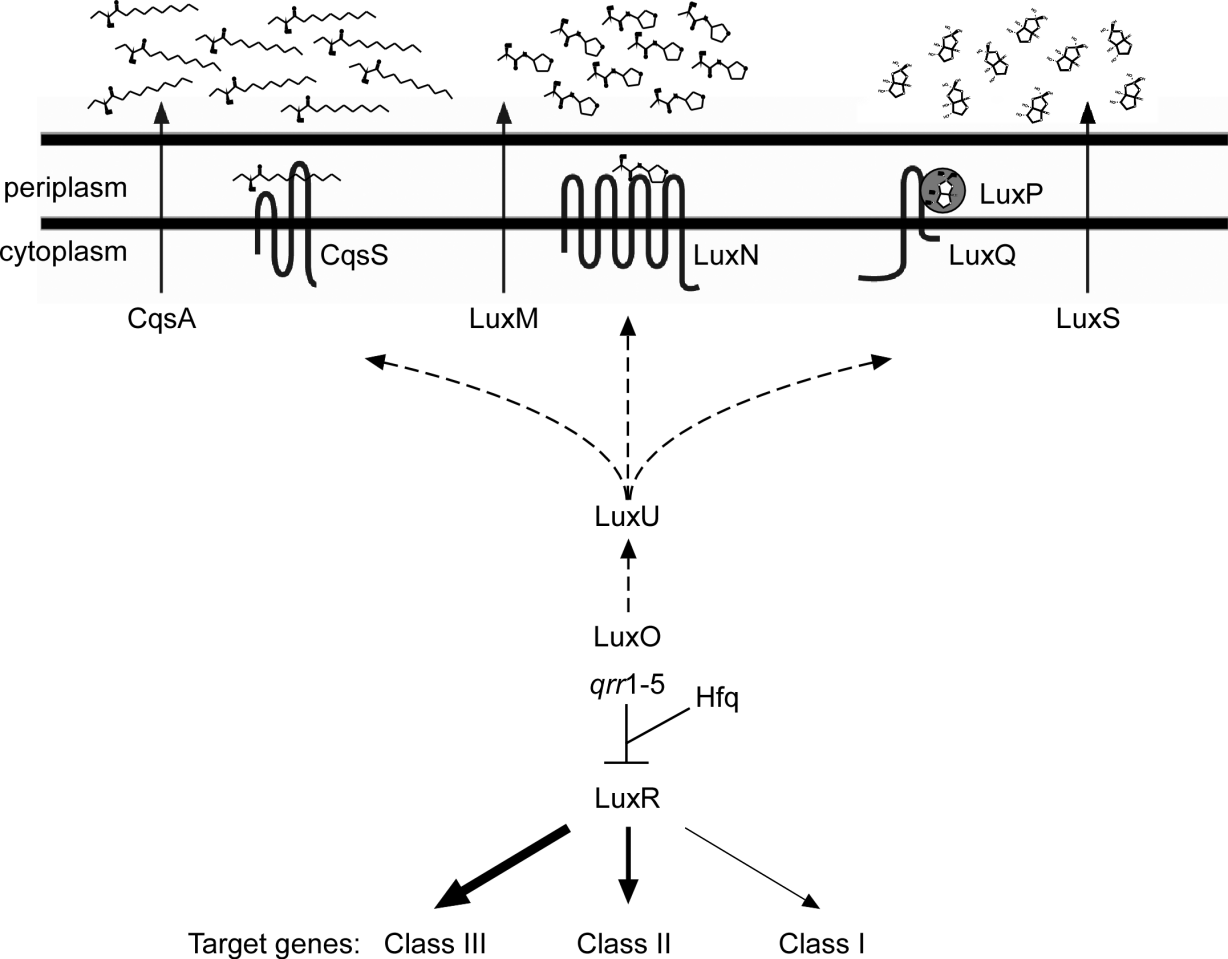
The *V. harveyi* quorum-sensing circuit. At high cell density, the three two-component sensors, CqsS, LuxN and LuxPQ, bind their respective AIs and act as phosphatases, resulting, via LuxU, in dephosphorylation of LuxO. This terminates *qrr*1–5 sRNA production and permits the translation of the master regulator, LuxR. Quorum-sensing target genes are classified according to their affinity for LuxR as indicated by arrow thicknesses. Class III genes have high affinity for LuxR, Class II genes have intermediate affinity and Class I genes have the lowest affinity. Dashed lines indicate the flow of phosphate at high cell density. The three AIs are CAI-1, HAI-1 and AI-2, and they are synthesized by CqsA, LuxM and LuxS respectively. Hfq is a sRNA chaperone.

Using a process called quorum sensing, bacteria communicate via secreted signal molecules called autoinducers (AI). As cell population density increases, AIs accumulate and trigger population-wide changes in the expression of genes involved in processes including motility, biofilm formation, virulence, type III secretion and bioluminescence (Waters and Bassler, 2005). The marine bacterium, *Vibrio harveyi*, makes and responds to at least three different AIs; HAI-1 (3-hydroxybutanoyl homoserine lactone) (Cao and Meighen, 1989), CAI-1 [(*S*)-3-hydroxytridecan-4-one] (Higgins *et al*., 2007) and AI-2 [(2*S*,4*S*)-2-methyl-2,3,3,4-tetrahydroxytetrahydrofuran-borate] (Chen *et al*., 2002). HAI-1, CAI-1 and AI-2 are suggested to mediate intra-species, intra-genera and inter-species cell–cell communication respectively (Henke and Bassler, 2004a). *V. harveyi* detection of these AIs requires three cognate two-component sensors that function in a phosphorelay cascade that impinges on the phosphorylation state of the response-regulator protein LuxO (Fig. 1) (Bassler *et al*., 1993; 1994; Freeman *et al*., 2000; Lilley and Bassler, 2000; Miller *et al*., 2002; Henke and Bassler, 2004a). At low cell density, phospho-LuxO activates transcription of genes encoding five small RNAs (*qrr*1–5) that act to destabilize the *luxR* transcript, preventing production of the global quorum-sensing regulatory protein, LuxR (Lenz *et al*., 2004; Tu and Bassler, 2007). Increasing AI concentration reverses the phosphorelay, depleting phospho-LuxO, which leads to termination of *qrr* expression and a corresponding increase in LuxR production. LuxR, directly or indirectly, controls the expression of genes in the quorum-sensing regulon (Waters and Bassler, 2006).

In many Gram-negative bacteria, e.g. *Vibrio fischeri*, *Pseudomonas aeruginosa* and *Agrobacterium tumefaciens*, quorum sensing is mediated by a LuxIR-type system in which a LuxI-type enzyme produces a homoserine lactone AI molecule that is subsequently bound by a cognate cytoplasmic DNA-binding protein of the LuxR type (Miller and Bassler, 2001). However, these LuxR proteins are not related to *V. harveyi-*type LuxR proteins. Rather, *V. harveyi* LuxR is a member of the TetR protein superfamily: repressor proteins that share conserved N-terminal helix–turn–helix DNA binding domains (Jobling and Holmes, 1997). TetR-type proteins act as dimers and recognize DNA operator sequences possessing dyad symmetry. Usually, upon binding a cognate small-molecule ligand, TetR-type proteins release DNA, allowing RNA polymerase access to the target promoter (Ramos *et al*., 2005). The classic example is TetR (e.g. in *Escherichia coli*), which represses its own expression and that of *tetA*, the gene encoding the tetracycline exporter that confers resistance to the drug. When tetracycline enters the cell, it is bound by TetR, inducing a conformational change that inactivates TetR DNA binding capability (Hillen and Berens, 1994). QacR, CprB and EthR are also canonical TetR proteins that have been studied extensively and behave analogously to TetR (Ramos *et al*., 2005). The TetR-type protein BetI, on the other hand, possesses the conserved helix–turn–helix domain, but it binds to DNA both in the absence and presence of its ligand, choline (Rkenes *et al*., 1996).

Recently, the crystal structure of the TetR-type protein HapR from *Vibrio cholerae* was solved (De Silva *et al*., 2007). HapR is 71% identical to *V. harveyi* LuxR, and HapR plays the same role in *V. cholerae* as LuxR does in *V. harveyi*, i.e. LuxR/HapR is the master regulator of the quorum-sensing response (Zhu *et al*., 2002; Henke and Bassler, 2004b; Lenz *et al*., 2004). The HapR structure suggests the presence of a ligand binding pocket; however, no small molecule has been identified that modulates HapR activity (De Silva *et al*., 2007). We anticipate that LuxR/HapR represses transcription via a mechanism characteristic of other TetR-type proteins, by interfering directly with RNA polymerase. Alternatively, LuxR/HapR could repress transcription by obstructing DNA binding of ancillary activator proteins. HapR is known to function by this latter mechanism in at least one case, at the *V. cholerae aphA* promoter. Specifically, *aphA* transcription is activated by the combined action of the transcription factors Lrp and VpsR, both of which are antagonized by HapR (Lin *et al*., 2007).

The *V. harveyi* LuxR protein is the founding member of a family of homologous proteins that exist in all Vibrio species. In every species examined, the LuxR-type protein controls a variety of behaviours. This facet sets the LuxR-type proteins apart from other TetR-type proteins, which generally control only their own transcription and that of an adjacent gene or operon (e.g. *tetR/tetA*). Another interesting feature of *V. harveyi* LuxR that makes it unlike other characterized TetR-type proteins is that while it clearly represses expression of some genes, LuxR also functions as an activator. For example, LuxR is a direct activator of the *lux* operon (encoding luciferase) (Martin *et al*., 1989; Showalter *et al*., 1990; Miyamoto *et al*., 1994) and several other quorum-sensing target genes (Waters and Bassler, 2006). A few other TetR-type proteins have been suggested to activate gene expression; however, no mechanism has been described to explain this phenomenon (Alatoom *et al*., 2007; Chatterjee *et al*., 2007; Hirano *et al*., 2008). LuxR-directed activation of gene transcription could occur through direct recruitment of RNA polymerase or through DNA bending. LuxR could also interfere with other transcription factors, similar to the role HapR plays at the *aphA* promoter, but resulting in gene activation rather than repression.

Defining the mechanism of *V. harveyi* LuxR regulation is complicated by the fact that LuxR controls genes differentially with respect to the discrete AI inputs (Fig. 1). As mentioned, each AI encodes unique information regarding the relatedness of the vicinal population, and *V. harveyi* commonly exists at different cell population densities and in a variety of bacterial consortia, exposing it to different combinations of AIs. Accordingly, these different signal mixtures lead to different cytoplasmic concentrations of LuxR (Tu and Bassler, 2007). LuxR availability, in turn, defines which promoters are bound and activated, or repressed, under a given condition. To date, we have identified three classes of LuxR-regulated target genes. Class I genes require the highest concentration of LuxR protein for regulation because their promoters have the lowest affinity for LuxR. Class II genes respond to intermediate concentrations of LuxR. Class III promoters have the highest affinity for LuxR and thus require the lowest level of LuxR for regulation (Waters and Bassler, 2006). In terms of temporal expression, Class III genes are activated/repressed first, followed by Class II, and then Class I genes. While our knowledge of the *V. harveyi* quorum-sensing regulon is incomplete, all of the directly LuxR-activated promoters that we have identified are Class I genes. In contrast, directly LuxR-repressed genes are members of all three classes. We presume that a particular promoter's affinity for LuxR is determined by the similarity of its binding site or sites to a consensus LuxR binding sequence and thus defines whether the promoter is Class I, II or III.

To investigate how LuxR directly regulates genes with differing affinities, and to examine how LuxR can act as both an activator and a repressor, we determined the LuxR DNA recognition sequence using protein binding microarrays (PBMs) (Bulyk *et al*., 2001; Mukherjee *et al*., 2004; Berger *et al*., 2006). The information garnered from the PBMs coupled with genomic sequence scanning and development and application of several bioinformatic algorithms allowed us to define the optimal LuxR binding site, as well as to identify potential LuxR binding sites upstream of putative conserved open reading frames (ORFs). To explore LuxR-DNA binding specificity, we used site-directed mutagenesis to generate mutations in LuxR-binding DNA sequences and analysed the consequences both *in vitro* and *in vivo*. LuxR binds a 21 bp operator with dyad symmetry. The critical bases for binding in each half-site are independent of one another, leading to context-dependent DNA binding affinity and specificity. In a proof-of-principle analysis, we scanned the *V. harveyi* genome for putative LuxR binding sites and tested several of the candidate promoters for LuxR regulation. Indeed, the candidate genes are controlled by LuxR, confirming that our approach allows us to identify novel members of the *V. harveyi* quorum-sensing regulon. We now intend to use this strategy to identify the entire set of LuxR-regulated genes.

## Results

### PBM

The LuxR protein was engineered with an N-terminal glutathione S-transferase (GST) tag and purified. Electrophoretic mobility shift assays (EMSA), using a fragment of the *aphA* promoter known to bind LuxR, confirmed that our purified GST-LuxR specifically bound DNA *in vitro*. In addition, using bioluminescence as the readout, we determined that the GST-LuxR complemented a *V. harveyi luxR* null mutant, showing that the engineered protein also functions *in vivo* (data not shown). To characterize LuxR's DNA binding specificity, universal PBMs containing approximately 44 000 double-stranded DNA sequences were designed to contain all possible 10-mer sequence variants. Importantly, all contiguous and gapped 8-mers, including all possible 4-gap-4 variants with gaps of up to 20 nucleotides, were covered 32 times each in the synthetic sequences used to construct these arrays (Berger *et al*., 2006).

**Fig. 2:**
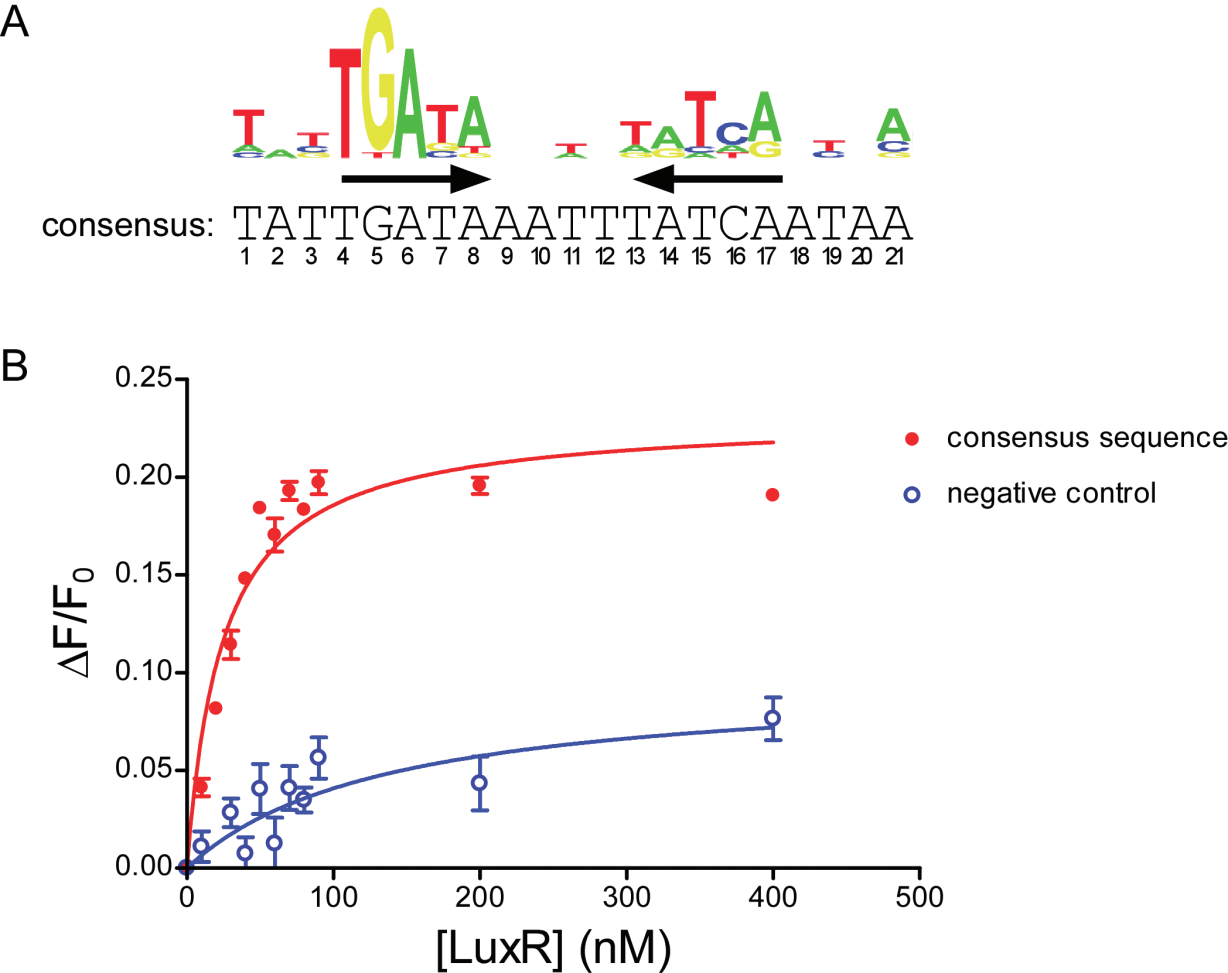
LuxR binds a 21 bp operator with dyad symmetry. A. The PWM derived from the PBM data. The consensus sequence was generated with the most frequent base at each position from the PWM. Inverted arrows represent the dyad symmetry. B. DNA binding curves for LuxR binding to the PWM consensus sequence (red circles) or a randomized negative control sequence (blue circles). The fractional change in anisotropy, where Δ*F* = *F* − *F*_0_, is plotted against the concentration of LuxR (nM). Error bars represent the standard deviation for three independent binding reactions.

The PBMs were incubated with GST-LuxR, and bound protein was subsequently detected with a fluorescently labelled anti-GST antibody. We used the program MultiFinder (Huber and Bulyk, 2006) to identify and align over-represented motifs in the 50 best LuxR-bound sequences on the PBM, according to normalized fluorescence intensity. The resulting position weight matrix (PWM) model of the binding site describes the preferred nucleotide at each position in a probabilistic manner. As shown in Fig. 2A, MultiFinder identified a 21 bp binding site with dyad symmetry (arrows) flanking a 4 bp region of non-specific sequence. This site is similar to those defined for other TetR-type transcription factors (Ramos *et al*., 2005). To test the validity of the PBM results, we synthesized a 31 bp fluorescein-labelled double-stranded DNA fragment containing the putative 21 bp LuxR consensus binding sequence (see Fig. 2A) and incubated it with LuxR. Binding was measured *in vitro* using fluorescence anisotropy (Heyduk *et al*., 1996; Head *et al*., 1998). Figure 2B shows that following incubation of LuxR with a DNA fragment containing the consensus sequence (red circles), a significant increase in anisotropy occurred. By contrast, LuxR did not bind to a DNA fragment of the same size containing a random sequence (blue circles). The apparent dissociation constant for the consensus sequence binding reaction is 24 ± 4 nM (Table 1).

**Table 1:** Dissociation constants for LuxR at various binding sites.

	*K*_d_ (nM)	Standard deviation (nM) for *n* = 3
Consensus sequence	24	4
Negative control	136	63
*qrgB* wild-type	27	3
A2C	27	4
A6C	44	5
A17C	43	6
A2C A17C	242	46
*qrr4* wild-type	35	4
A6C	68	11
T15C	27	4
A17C	38	5
A6C A17C	112	27
VP0057/8	47	8
VP0944/5	30	7
VPA0197/8	68	11
VPA0226/7	73	12
VPA0649	30	8

In order to scan and score genomic DNA sequences for potential LuxR binding sites, we developed a two-layered approach that uses the PWM as the first layer, and a support vector machine (SVM) as a second layer (see Supporting information). Potential 21 bp sites identified by the PWM were further evaluated using the SVM model, which takes into account the context of the entire site, and automatically allows for codependencies between different positions as well as for different weights for the individual positions. In practice, the higher the SVM score, the more a particular sequence resembles those sequences on the PBM that bound most tightly to GST-LuxR. The two-layered approach including the SVM is much more selective for actual binding sites than the PWM alone. When tested on the raw PBM data, the PWM alone was poor at recognizing true binding sites (those in sequences producing the highest fluorescence in the PBMs) at high confidence thresholds, and had a high false positive rate at low confidence thresholds. For example, at 95% confidence, the PWM recognized only 8% (7/88) of the bound sequences on the PBM, with a 0.002% (2/83 790) false positive rate. At 85% confidence, the PWM recognized 99% (87/88) of the bound sequences, but with a 2.5% (2053/83 790) false positive rate (the false positive rate is for returning a false positive 21 bp sequence anywhere within the 36 bp of variable sequence contained within each 60 bp probe on the PBM). By contrast, our two-layered PWM/SVM model performed much better on the PBM data. For example, one of our best performing PWM/SVM models recognized 99% of the bound sequences with a 0.05% false positive rate (see Table S1).

It is clear from the dyad symmetry of the LuxR consensus binding site that LuxR, like other TetR-type proteins, binds DNA as a dimer. In order to learn more about the mechanism of LuxR binding from the PBM data, we used the SVM layer of the bioinformatics algorithm to probe the mechanism of LuxR binding. Starting with the consensus binding site obtained by the PWM (Fig. 2A), which is also the site with the highest score according to our SVM algorithm, we calculated SVM scores for all possible single base substitutions in the 21 bp sequence. The five most deleterious mutations were G5T, A6C, A8G, T15C and A17C, confirming the importance of the regions of dyad symmetry, while the five most benign mutations were T3C, T12A, A14G, A18G and A20T. To address whether it was possible to ‘rescue’ a deleterious mutation with a compensatory mutation elsewhere in the sequence, we selected the most deleterious mutation at each position and then obtained SVM scores for every possible additional substitution in the singly mutated sequences. Selecting for the highest scoring double mutants only revealed the same five most benign mutations of the consensus sequence, and these only marginally increased the SVM scores of their singly mutated parent sequences. In other words, we found no specific suppressors of the most deleterious mutations. Thus, according to our SVM model, for sequences similar to the consensus sequence, each base pair contributes independently to the overall binding affinity of LuxR.

### Predicting binding sites at known LuxR-regulated genes

**Fig. 3:**
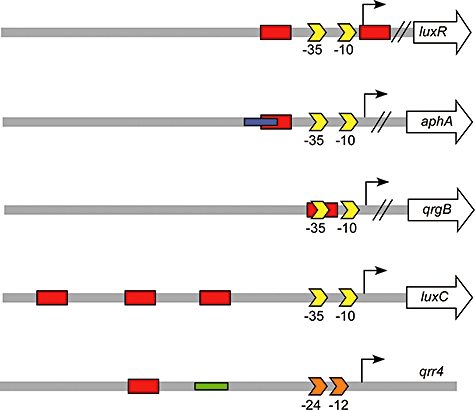
LuxR binding sites upstream of quorum sensing-regulated genes. The two-layered PWM-SVM approach confirmed the known LuxR binding sites upstream of *luxR*, *aphA* and *luxC* and predicted sites upstream of *qrgB* and *qrr*4 (positions on DNA are shown to scale). LuxR binding sites are indicated by red boxes, the VpsR binding site at *aphA* is shown in blue, and the LuxO-P binding site at *qrr*4 is shown in green. σ^70^ −35 and −10 promoter sequences are shown as yellow arrows, while the −24 and −12 sequences at the *qrr*4 σ^54^-dependent promoter are indicated by orange arrows.

To test our ability to identify LuxR binding sites, we used the above two-layered procedure to scan the DNA sequences upstream of target genes with well-characterized direct regulation by LuxR, as a proof-of-principle. We found putative binding sites present in known LuxR binding regions upstream of *aphA, luxC* and *luxR* (Fig. 3, red boxes). Notably, previous binding studies with LuxR upstream of *luxC* relied on footprinting and EMSA analyses and could only confine LuxR binding to a rather large region of promoter DNA (Miyamoto *et al*., 1994). Our analysis pinpoints the exact LuxR binding sites within this region. We also scanned the sequences upstream of other genes in the quorum-sensing regulon that had not previously been analysed for LuxR control. We confirmed that LuxR binds upstream of the Class III gene *qrgB,* encoding a GGDEF domain-containing protein involved in cyclic di-GMP synthesis (Waters and Bassler, 2006; Waters *et al*., 2008). Surprisingly, we also identified putative LuxR binding sites upstream of the genes encoding the sRNAs *qrr*2, *qrr*3 and *qrr*4 (Fig. 3, red box shows the site for *qrr*4), suggesting a role for LuxR in the regulation of quorum-sensing sRNA gene expression. To examine whether LuxR controls *qrr* expression, we introduced a *qrr*4–*gfp* promoter fusion into *E. coli* carrying a chromosomal copy of a phosphomimetic LuxO variant, *luxO* D47E. Phospho-LuxO or a mimetic is an absolute requirement for *qrr* gene expression (see Fig. 1) (Svenningsen *et al*., 2008). Indeed, LuxR enhances phospho-LuxO-dependent activation of *qrr*4 transcription (discussed further below), indicating that an internal feedback loop exists in the *V. harveyi* quorum-sensing circuit in which LuxR activates transcription of the *qrr* genes.

To test the sequence requirements for binding of LuxR to DNA *in vivo*, we chose one LuxR-activated promoter and one LuxR-repressed promoter for further examination. We selected the promoters for *qrr*4 (activated) and *qrgB (*repressed) because each appeared to contain only a single LuxR binding site with strong similarity to the consensus binding sequence. We did not study the *luxC* and *luxR* promoters because both are Class I targets, suggesting that they have low affinity for LuxR (Waters and Bassler, 2006), and both contain multiple LuxR binding sites, which would have complicated our analysis. We also did not further examine the *aphA* promoter because, as mentioned, HapR binding to that promoter has already been well characterized and other factors are involved (Kovacikova and Skorupski, 2002; Lin *et al*., 2005; 2007). We note that in some *V. cholerae* isolates there exists a naturally occurring mutation (G-77T) that abolishes HapR regulation of *aphA* (Lin *et al*., 2005). This transversion corresponds to position 5 of our consensus site, which is a G in 96% (77% if reverse complements are taken into account) of the bound sequences on the PBMs, indicating that a G in this position is critical for both HapR and LuxR binding.

**Fig. 4:**
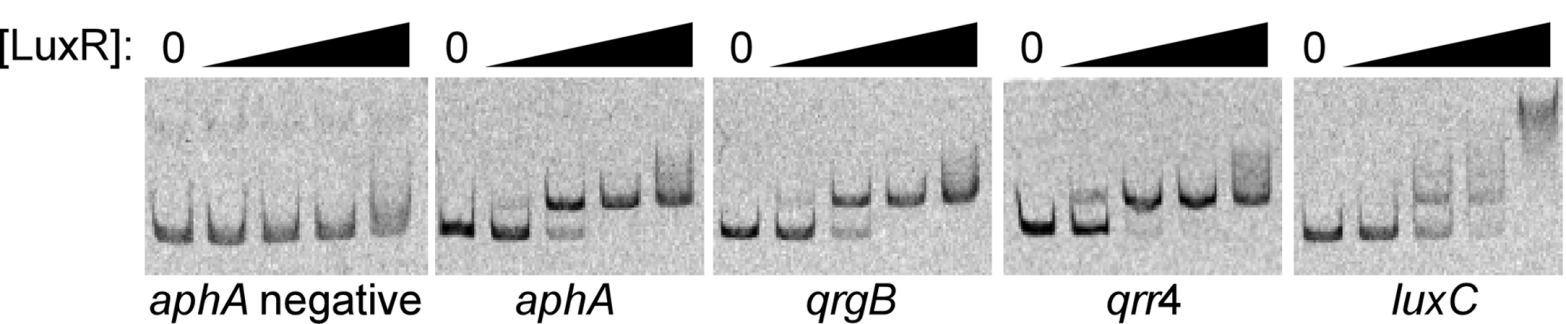
LuxR binds one site within the *qrgB* and *qrr*4 promoters. LuxR binding to the *aphA*, *qrgB*, *qrr*4 and *luxC* promoters was determined by EMSA with increasing concentrations of LuxR. The first panel is a negative control and shows a DNA fragment containing a region upstream of *aphA* that does not bind LuxR. Lane 1 in each panel contains no LuxR, and lanes 2–5 contain 10, 50, 100 and 250 nM LuxR respectively.

To confirm that the *qrr*4 and *qrgB* promoters each contain only a single LuxR binding site, we performed EMSA with DNA fragments containing the 500 bp upstream of each transcription start site. As a control, we show that there was no shift of a DNA fragment containing an upstream region of the *aphA* promoter that is known not to be bound by LuxR (Fig. 4) (Svenningsen *et al*., 2008). By contrast, the upstream region of *aphA* containing the reported LuxR binding was shifted by LuxR. This region is known to bind a single dimer of LuxR (Lin *et al*., 2005). LuxR incubation with the *qrgB* and *qrr*4 fragments resulted in shifts identical to that produced by LuxR binding at the *aphA* fragment, confirming that LuxR binds to one site in these regions. In the right-most panel of Fig. 4, by way of comparison, we show that LuxR super-shifts the *luxC* promoter fragment that is predicted to contain multiple binding sites.

### Computational and experimental mutagenesis of the LuxR binding site

**Fig. 5:**
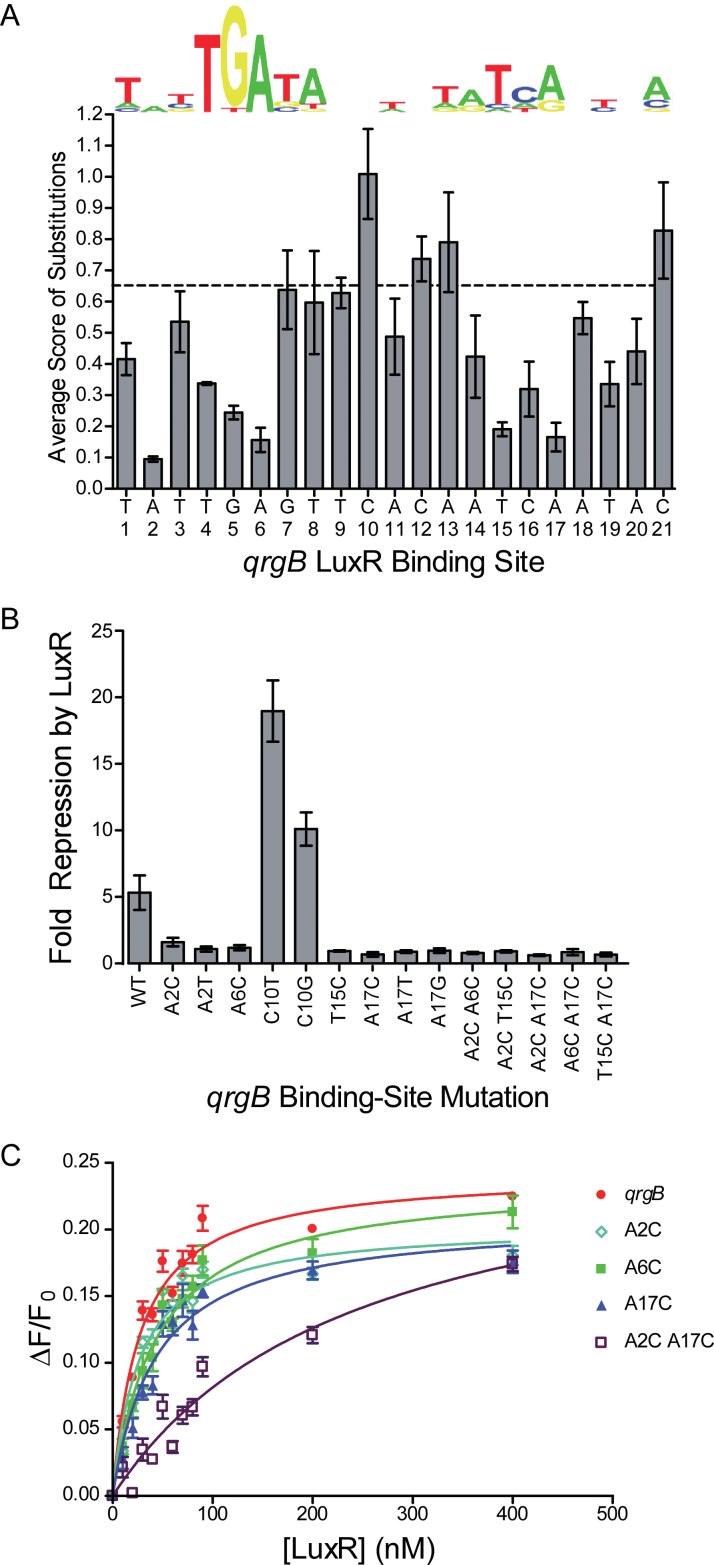
LuxR binds and regulates *qrgB*. A. The consensus PWM for the LuxR binding site is shown for comparison with the actual binding site in the *qrgB* promoter. The wild-type binding site is scored at 0.65 (dashed line). The average SVM score for the three substitutions at each base is presented versus the location in the binding site. Error bars indicate the standard deviation of the mean score for mutations at each position. B. *In vivo* repression of *qrgB–gfp* expression is shown for the wild-type promoter, single- and double-point mutants. Measurements were made in triplicate, and fold repression calculated as the ratio LuxR^-^/LuxR^+^. Error bars represent the standard deviation of the ratio, calculated via the formula for propagation of error. C. DNA binding curves for LuxR binding to the wild-type *qrgB* binding site (red), *qrgB* A2C (light blue), *qrgB* A6C (green), *qrgB* A17C (dark blue) and *qrgB* A2C, A17C (purple). The fractional change in anisotropy is plotted against the concentration of LuxR (nM). Error bars represent the standard deviation of three independent binding reactions.

**Fig. 6:**
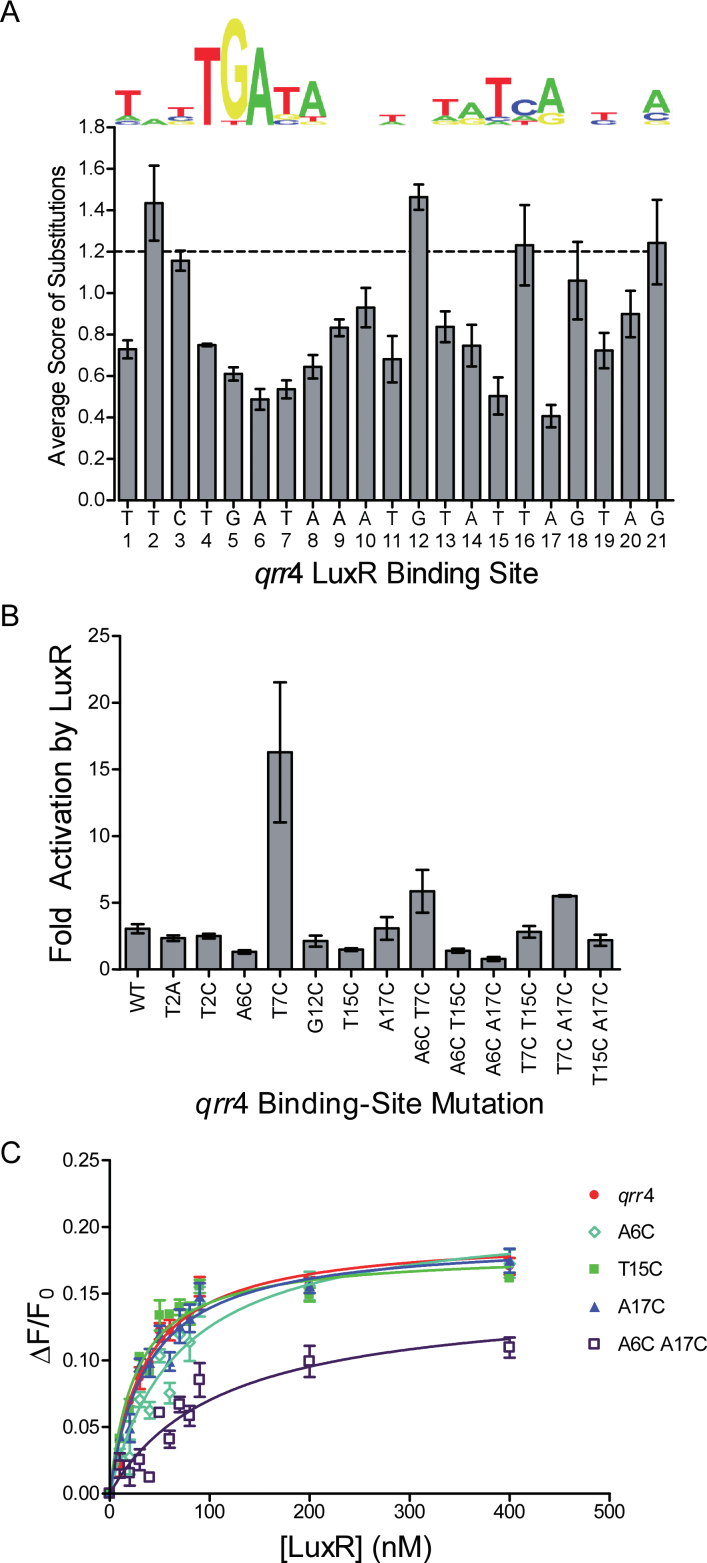
LuxR binds and regulates *qrr*4. A. The consensus PWM for the LuxR binding site is shown for comparison with the actual binding site in the *qrr*4 promoter. The wild-type binding site is scored at 1.2 (dashed line). The average SVM score for the three substitutions at each base is presented versus the location in the binding site. Error bars indicate the standard deviation of the mean score for mutations at each position. B. *In vivo* activation of *qrr*4–*gfp* expression by LuxR is shown for the wild-type promoter, single- and double-point mutants. Fold activation was calculated as the ratio LuxR^+^/LuxR^-^. Error bars represent the standard deviation of the mean ratio from two independent experiments. C. DNA binding curves for LuxR binding to the wild-type *qrr*4 binding site (red), *qrr*4 A6C (light blue), *qrr*4 T15C (green), *qrr*4 A17C (dark blue) and *qrr*4 A6C, A17C (purple). The fractional change in anisotropy is plotted against the concentration of LuxR (nM). Error bars represent the standard deviation of three measurements.

To investigate the requirements for LuxR binding at the *qrr*4 and *qrgB* promoters, we analysed the two corresponding LuxR binding sites by *in silico* mutagenesis. All possible single-nucleotide substitutions were made at each of the 21 positions in the *qrgB* and *qrr*4 LuxR binding sites, and the resulting sequences scored for the likelihood of LuxR binding using our SVM model, as shown in Fig. 5A (*qrgB*) and Fig. 6A (*qrr*4). In these panels, bars with lower heights (i.e. lower SVM scores) indicate sites where base changes are predicted to have the most deleterious effects on LuxR binding. In the case of LuxR binding at *qrgB*, we found the most deleterious alterations to be at positions 2, 6, 15 and 17 (Fig. 5A) (for comparison, recall that the most deleterious mutations in the consensus sequence are at positions 5, 6, 8, 15 and 17, indicating that as the sequences diverge sufficiently far from the consensus sequence, some context dependence arises for the contribution of each site to LuxR binding). To test the *qrgB* predictions, we engineered the A2C, A2T, A6C, T15C, A17C, A17T and A17G mutations into the *qrgB* promoter–*gfp* fusion construct and measured repression by LuxR in *E. coli* (Fig. 5B). The wild-type *qrgB* promoter is repressed fivefold by LuxR. The A2C mutation reduced repression by LuxR to 1.6-fold, and all of the other mutations completely abolished LuxR-dependent repression. We also engineered sites containing combinations of the above mutations into the *qrgB–gfp* reporter construct. As expected, LuxR did not repress any of the double-mutant constructs (Fig. 5B). Our computational mutagenesis predicted that bases located from positions 9–12, i.e. between the inverted repeats, were of minimal importance as *in silico* substitutions of these nucleotides did not significantly reduce the SVM scores (Fig. 5A). Consistent with this prediction, when we mutated a base in this intervening region (C10T and C10G), we observed repression by LuxR to the same levels as for the wild-type sequence (Fig. 5B) (in Fig. 5B, LuxR repression of the C10T and C10G constructs appears stronger than for wild type only because in the absence of LuxR, the basal expression of these two constructs is higher than that of the wild-type construct).

To determine whether the above alterations in LuxR control of *qrgB* transcription were due to the inability of LuxR to bind to the mutated binding sites, we used fluorescence anisotropy to analyse LuxR binding to the wild-type *qrgB* site as well as to the A2C, A6C, A17C and the double A2C A17C-mutated sequences. All of the sequences were tested in the context of 33 bp 5′fluorescein-labelled DNA fragments (Fig. 5C). Table 1 summarizes the consequences of each mutation on the apparent LuxR binding affinity. In brief, LuxR bound to the wild-type *qrgB* sequence as avidly as it bound to the consensus fragment (*K*_d_ of 27 ± 3 nM). The construct containing the A2C mutation, which caused the most modest effect on repression *in vivo*, had the same dissociation constant for LuxR as the wild-type *qrgB* sequence did. The *K*_d_ was nearly double for the A6C and A17C sequences, and the binding constant for LuxR to the doubly mutated A2C A17C fragment was an order of magnitude higher than for the wild-type sequence.

Regarding LuxR binding at the *qrr*4 promoter, our computational analysis revealed a significantly higher overall SVM score for the LuxR binding site in the *qrr*4 promoter than for the *qrgB* promoter. Therefore, single-base mutations are predicted to have less drastic consequences at *qrr*4. Indeed, the SVM scores of the singly substituted *qrr*4 binding sites were all potentially consistent with continued LuxR binding (Fig. 6A). Nonetheless, we engineered single mutations into the *qrr*4 promoter–*gfp* fusion construct (A6C, T7C, T15C and A17C) at positions predicted to be the most important for LuxR binding. We also constructed combinations of pairs of these mutations. We introduced these fusions into the *E. coli* strain harbouring *luxO* D47E. Figure 6B shows that the wild-type *qrr*4 promoter is activated threefold by LuxR. Two single mutations, A6C and T15C, prevent LuxR activation as do double mutants containing these altered bases (A6C T15C and A6C A17C). The A17C single mutation retains wild-type LuxR activation, and the T7C mutation unexpectedly increases LuxR activation to 12-fold. Accordingly, all doubly mutated constructs that include the T7C transition remain activated by LuxR. Finally, the T15C A17C double mutant also remains capable of LuxR activation, presumably due to the contribution of the A17C change. Similar to our analysis of the *qrgB* promoter, we engineered a mutation at a central position of the *qrr*4 promoter, G12C, which was not predicted to be important for LuxR binding. As expected, LuxR continued to activate expression of this construct to wild-type levels.

Interestingly, according to our SVM model, single nucleotide substitutions at position 2 in the *qrr*4 LuxR binding site should have no consequence for LuxR regulation, despite the critical role of this nucleotide for LuxR binding at the *qrgB* promoter. To test this prediction, we made the T2C and T2A substitutions in the *qrr*4 binding site and analysed their regulation by LuxR (Fig. 6B). Neither of these mutations affected LuxR activation at the *qrr*4 promoter. From these data, we conclude that although LuxR clearly exhibits sequence specificity, the sequence requirements depend on the context of the complete binding site.

Using fluorescence anisotropy, we determined the affinity of LuxR *in vitro* for the wild-type *qrr*4 binding site, and the A6C, T15C, A17C and double A6C A17C-mutated sites. As shown in Fig. 6C and summarized in Table 1, LuxR bound the wild-type sequence with an apparent *K*_d_ of 35 ± 4 nM. Mutating T15C or A17C had little or no effect on the apparent *K*_d_, with LuxR binding slightly better to the T15C fragment. The A6C mutation increased the *K*_d_ twofold, and the double A6C A17C mutant sequence had a *K*_d_ of 112 ± 27 nM, roughly fourfold that of the wild-type sequence. We note that our *in vitro* binding results are not absolutely correlative to *in vivo* regulation. Likely, other factors are involved *in vivo* that modulate LuxR's activity.

### Genome-wide scan to predict novel LuxR targets

**Fig. 7:**
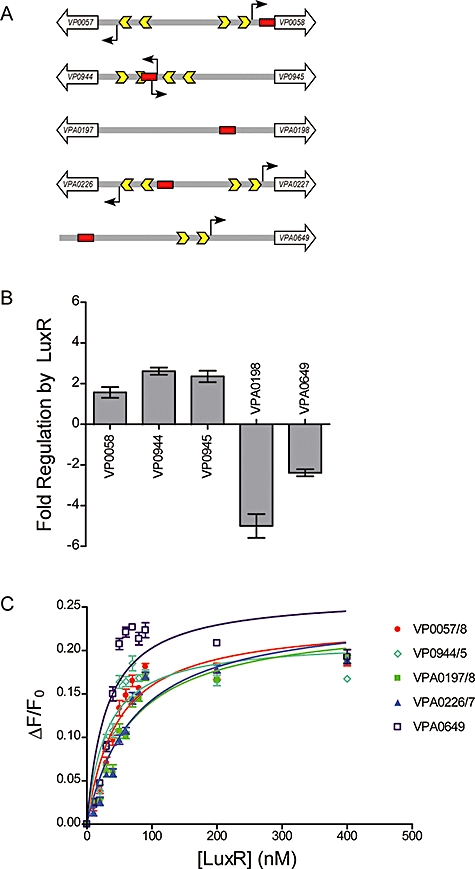
Predicted genomic targets of LuxR. A. Five promoter regions containing predicted LuxR binding sites (red boxes) identified in the *V. harveyi* genome are shown. Transcriptional start sites were identified, where possible, with 5′RACE (black arrows), and σ^70^ −35 and −10 promoter sequences are shown as yellow arrows. No transcriptional start site could be identified for VPA0197 or VPA0198. B. *gfp* expression from predicted promoter fusions (shown in A) in *E. coli*. Fold regulation by LuxR is represented as positive values for activated genes and negative values for repressed genes. Genes that showed no expression are not included in the panel. Measurements were made in triplicate, and fold repression calculated as the ratio LuxR^-^/LuxR^+^ for repressed promoters and LuxR^+^/LuxR^-^ for activated promoters. Error bars represent the standard deviation of the ratio, calculated via the formula for propagation of error. C. *In vitro* LuxR binding to the predicted binding sites from A. Binding curves are shown for VP0057/8 (red), VP0944/5 (light blue), VPA0197/8 (green), VPA0226/7 (dark blue) and VPA0649 (purple). The fractional change in anisotropy is plotted against the concentration of LuxR (nM). Error bars represent the standard deviation of three independent binding reactions.

Multiple screens have been performed to identify the *V. harveyi* genes regulated by quorum sensing, and these have yielded over 50 genes in the regulon, although only 10 of these genes are controlled directly by LuxR (Waters and Bassler, 2006). We have no evidence to suggest that the screens have been saturated, so we hoped to exploit our findings here to extend our understanding of the *V. harveyi* quorum-sensing regulon. We scanned the *V. harveyi* genome for LuxR binding sequences using our two-layered PWM/SVM scoring system. We identified 36 sites and selected five of the highest scoring candidate binding sites for testing. These sites (Fig. 7A) were chosen because they were located within 300 bp of a predicted ORF, and they were strongly conserved (see Supporting information) between *V. harveyi* and the closely related bacterium, *Vibrio parahaemolyticus*, suggesting that the putative binding sites are functionally relevant [note: nomenclature refers to annotation from the *V. parahaemolyticus* genome (JCVI-CMR) because the *V. harveyi* genome has not yet been annotated].

To determine the effect of LuxR on the expression of these candidate genes *in vivo*, a 500 bp region containing each putative promoter (Fig. 7A) was cloned and fused to *gfp*. Four of these sites were positioned between two divergent ORFs (Fig. 7A), so we fused the putative promoter to *gfp* in both orientations, making a total of nine constructs. The constructs (named for the ORF transcribed in the same direction as the promoter fusion) were tested for LuxR regulation in *E. coli* as described for the *qrgB* promoter fusions. Five of the nine promoter fusions are measurably expressed in *E. coli* and all five are clearly regulated by LuxR. Figure 7B shows that VP0058, VP0944 and VP0945 are activated 1.6-fold, 2.6-fold and 2.4-fold by LuxR respectively, while LuxR represses VPA0198 and VPA0649 5-fold and 2.3-fold respectively. VPA0057, VPA0197, VPA0226 and VP0227 were not measurably expressed, so the effect of LuxR could not be determined.

We assessed LuxR binding to each of the predicted sites *in vitro* by fluorescence anisotropy*.* Indeed, LuxR bound to all of the candidate sites with reasonable affinity (Fig. 7C and Table 1). Specifically, LuxR bound the sites upstream of VP0944/5 and VPA0649 with similar affinity to the consensus sequence (*K*_d_ of 30 ± 7 nM and 30 ± 8 nM respectively). The site upstream of VP0057/8 was bound by LuxR with a slightly lower affinity (*K*_d_ of 47 ± 8 nM), and the sites upstream of VPA0197/8 and VPA0226/7 were bound with the lowest affinity (*K*_d_ of 68 ± 11 nM and 73 ± 12 nM respectively).

## Discussion

LuxR, a TetR-type DNA-binding protein, is the master regulator of quorum sensing in the marine bacterium *V. harveyi*, one of the first two bacterial species shown to use quorum sensing and the first bacterium discovered to communicate across species boundaries. Quorum sensing is intensively studied in *V. harveyi* because the quorum-sensing network is viewed as a paradigm for understanding how sensory information is integrated, processed and transduced to control gene expression. Nonetheless, despite numerous screens, the complete *V. harveyi* quorum-sensing regulon is not known; in particular, we surmised that additional targets directly regulated by LuxR existed and remained to be identified. To discover these targets, we performed a PBM study to characterize the DNA binding specificity of LuxR. The resulting quantitative *in vitro* binding results allowed us to train a two-layered bioinformatics algorithm capable of identifying LuxR binding sites upstream of genes known to be directly regulated by LuxR, and capable of scanning the *V. harveyi* genome for additional, previously unknown LuxR binding sites. Five novel genomic LuxR binding sites were identified using this test strategy, and LuxR binding to them was confirmed with *in vitro* studies. This analysis puts us in the position to now identify all the genes in the regulon. While our model successfully predicts binding by LuxR, it does not make any predictions about promoter strength or whether other factors are required for transcription *in vivo*. We suspect that *in vivo*, quorum-sensing information is integrated with other sensory cues for precise control of gene expression.

*Vibrio harveyi* LuxR is the founding member of a family of homologous proteins (e.g. OpaR, SmcR, VanT, HapR and LitR) in different Vibrio species. While analogous quorum-sensing pathways feed information to LuxR and its homologues (Fig. 1), the downstream regulon in each species diverges, allowing each communication circuit to carry out unique species-specific biology. The LuxR-type regulons are typically large, containing ∼100+ genes. The complexity and plasticity of these Vibrio quorum-sensing regulons requires a means to identify targets of LuxR-type proteins on a genome-wide scale. In the present work, we have demonstrated that a combination of PBM studies and bioinformatics provides a practical way to identify LuxR binding sites. PBM studies are particularly valuable for transcription factors, like LuxR, whose verified direct targets are too few in number to usefully train an algorithm to search for new binding sites. Furthermore, an important advantage of PBM studies is that they provide both a large number of positive examples of binding sites, and a large number of similar but negative examples. These two types of examples allow accurate discrimination of true binding sites from similar but non-binding sites, even for proteins such as LuxR with context-dependent binding specificities. Indeed, without the large number of both positive and negative examples provided by the PBM to train the final SVM layer of our bioinformatics algorithm, it would have been impossible to reduce the number of genome-wide false positives to a level practical for experimental verification. We note that an alternative hidden markov model has been successfully employed to identify binding sites of SmcR, the *Vibrio vulnificus* LuxR homologue. The hidden markov model was trained on 18 experimentally defined binding sites and used in a genome-wide search to identify new SmcR-regulated targets. Ten targets were experimentally verified to be regulated by SmcR. The 22 bp SmcR consensus sequence is similar to the 21 bp LuxR consensus sequence we find here. The SmcR and LuxR consensus sequences diverge in the outermost bases (Lee *et al*., 2008).

In this and previous studies we and others have identified several genes for which LuxR functions directly as an activator. The majority of characterized TetR-type proteins are only known to act as repressors. This raises the question of whether some specific selection has driven LuxR to evolve a positive regulatory role. Typically, expression of genes under the control of a TetR-type repressor is activated when the TetR-type repressor binds a small-molecule ligand and releases the DNA. It seems possible that an ancestral LuxR originally carried out quorum sensing in this mode, de-repressing genes in response to binding of a small AI molecule that could enter the cytoplasm. In fact, AI binding by cognate cytoplasmic transcription factors is a common mode of operation in other quorum-sensing systems, such as those of *V. fischeri*, *P. aeruginosa*, *A. tumefaciens* and *Chromobacter violaceum*. In contrast, the *V. harveyi*-type LuxR and its homologues in other Vibrio species are regulated indirectly by extracellular AIs. To maintain the regulatory logic that high AI concentration leads to de-repression (activation) of genes, LuxR, in addition to being capable of repression, would have been required to add a positive regulatory function. It will be interesting to determine if an evolutionary intermediate can be found in which a *V. harveyi*-type LuxR homologue continues to function by direct binding of a small-molecule AI.

Our discovery of LuxR binding sites upstream of the sRNAs *qrr*2–4, along with experimental verification that LuxR directly positively regulates *qrr*4 (Fig. 4) as well as *qrr*2 and *qrr*3 (not shown), implies that LuxR's ability to function as an activator has been exploited for its own autoregulation. In *V. harveyi*, the *qrr* sRNAs negatively regulate LuxR by binding to and preventing translation of the LuxR message. The activation of transcription of these same quorum-sensing sRNAs by LuxR is suggested to accelerate the internal transition of cells from high- to low-cell-density modes, a potentially rapid event in the wild (Svenningsen *et al*., 2008). Later, after the transition from high- to low-cell-density mode is complete, a more modest rate of sRNA production is likely sufficient to repress quorum sensing. If so, direct activation of the *qrr* genes by LuxR implies that transcription of the *qrr* genes is scaled back precisely when LuxR levels fall and high transcription rates of the *qrr* genes are no longer required.

It remains an open question why quorum-sensing regulation of LuxR is achieved by sRNAs rather than at the level of transcription. A potential advantage of sRNA-based regulation is that LuxR mRNA lifetime is decreased, reducing the number of proteins translated per message (Levine *et al*., 2007). This mechanism prevents protein bursts, which in bacteria can amount to ∼100 translated proteins per message, even for nominally repressed genes. Our measurement of LuxR dissociation constants in the range of 30 nM via fluorescence anisotropy (Figs 2, 5 and 6 and Table 1) supports this interpretation. Within the typical cytoplasmic volume of a *V. harveyi* cell (≈1.6 μm^3^), 30 nM corresponds to ∼30 proteins. Therefore, if LuxR were only transcriptionally regulated, even a single mistimed message produced at low cell density could inappropriately trigger a downstream signalling cascade, with a potentially large cost to fitness.

In the future, we intend to exploit the combined PBM-bioinformatics approach reported here to expand the recognized quorum-sensing regulons of *V. harveyi* and other Vibrio species. Appreciation of these full regulons will help us understand individual versus group behaviours as well as the individual ecologies and survival strategies of these species, including the human pathogen *V. cholerae*.

## Experimental procedures

### Bacterial strains and conditions

*Vibrio harveyi* strain KM669 (*luxR*) was derived from wild-type BB120 (Bassler *et al*., 1997). *E. coli* strain S17-1 λ*pir* (de Lorenzo and Timmis, 1994) was used for conjugation, cloning and *gfp* expression experiments. *E. coli* strain KT1190 (*luxO* D47E) was derived from S17-λ*pir*. ElectroMAX DH10B (Invitrogen) and One Shot (Invitrogen) were used for cloning. *V. harveyi* strains were grown in Luria–Marine (LM) medium with aeration or on LM agar at 30°C. Plasmids were propagated in *E. coli* grown in Luria–Bertani LB broth with aeration or on LB agar at 37°C. *E. coli* used in *gfp* expression experiments was grown in LB broth with aeration at 30°C, except for experiments to test predicted target genes; those cultures were grown at 37°C. Antibiotics were used at the following concentrations: 100 μg ml^−1^ ampicillin, 100 μg ml^−1^ (for *E. coli*) or 250 μg ml^−1^ (for *V. harveyi*) kanamycin, 50 μg ml^−1^ polymyxin B and 10 μg ml^−1^ tetracycline. Bioluminescence was assayed from cultures of *V. harveyi* KM669 carrying pAP135 following growth overnight in LM broth with kanamycin and 100 mM IPTG.

### DNA manipulations

All PCR reactions used for cloning or to generate EMSA probes were performed with iProof DNA Polymerase (Bio-Rad). dNTPs, restriction endonucleases and T4 DNA ligase were obtained from New England BioLabs. Plasmids were introduced into *E. coli* either by transformation in 0.2 cm electroporation cuvettes (USA Scientific) with a Bio-Rad Micro Pulser or by conjugation followed by selection with appropriate antibiotics. Plasmids were introduced into *V. harveyi* by conjugation, and exconjugants were selected using the appropriate antibiotics and polymyxin B.

The *gst-luxR* overexpression construct used to purify GST-LuxR protein was engineered by amplifying the *luxR* ORF from *V. harveyi* genomic DNA and inserting it into pGEX4T-1 (GE Healthcare) between the EcoRI and XhoI restriction sites downstream of *gst*. The *gst-luxR* overexpression construct (pAP135) used to test complementation *in vivo* was generated by amplifying *gst-luxR* with AvrII and BglII overhanging ends and cloning the resulting fragment into pEVS143 (Dunn *et al*., 2006) using AvrII and BamHI, which placed *gst-luxR* under the control of the *P*_*TAC*_ promoter. To generate the promoter–*gfp* fusions for the analysis of predicted LuxR-controlled genes, 500 bp regions of each promoter were amplified from *V. harveyi* genomic DNA and inserted into pCMW1 (Waters and Bassler, 2006) between the SphI and SalI restriction sites.

### Protein purification

GST-LuxR overexpression was induced in *E. coli* BL21 cells with 100 μM IPTG for 6 h at 25°C. Cells were lysed in 25 mM Tris pH 7.5, 150 mM NaCl and 1 mM DTT. Lysates were loaded onto 4 ml of Glutathione-Uniflow resin (Clontech) and GST-LuxR was eluted with 50 mM Tris pH 7.5, 10 mM glutathione and 2 mM DTT. The protein in the eluate was further purified by anion-exchange, using a SourceQ 10/10 column. GST-LuxR-containing fractions (as determined by SDS-PAGE) were pooled and fractionated via a Superdex 75 16/60 gel filtration column.

Native LuxR protein overexpression was induced in *E. coli* BL21(DE3) codon plus cells with 0.4 mM IPTG for 5 h at 25°C. Cells were lysed at 4°C in 50 mM imidazole pH 8.0, 100 mM NaCl, 0.5 mM EDTA and 1 mM DTT by two passes through a continuous flow microfluidizer (Microfluidics). Cleared cell lysate was next passed through a Heparin column (Amersham Biosciences) and eluted with a gradient of NaCl (0.1 M–1 M). Fractions containing LuxR were concentrated using Centriprep YM-10 (Millipore) filters, dialysed against 50 mM imidazole pH 8.0, 150 mM NaCl, 0.5 mM EDTA and 1 mM DTT, and further fractionated by a Superdex200 16/60 gel filtration column (Amersham Biosciences).

### PBM and data analysis

The PBM experiments were performed essentially as described (Berger *et al*., 2006). Microarrays were synthesized by Agilent Technologies in the ‘4 × 44K’ format. GST-LuxR was diluted to a final concentration of 500 nM in separate 175 μl protein binding reaction mixtures, which were applied to the individual chambers of a four-chamber gasket coverslip. GST-LuxR was applied to two of the four chambers on both of our two separate PBM designs. Protein-bound arrays were labelled with Alexa488-conjugated anti-GST antibody (Sigma). Microarrays were scanned (GSI Lumonics ScanArray 5000) at multiple laser power settings, and raw image files were quantified using GenePix Pro version 6.0 software (Molecular Devices). Protein binding signal was normalized according to the observed relative amounts of double-stranded DNA and adjusted for spatial non-uniformities.

Microarray signal intensities were averaged for identical probes in separate chambers of the same slide. For each of the 8-mer patterns covered in our universal PBM, we were able to score the relative preference of LuxR for all sequence variants by calculating the median signal intensity over all probes containing a match to the 8-mer. We further calculated an enrichment score for each 8-mer, which could be combined across both array designs (Berger *et al*., 2006).

We attempted to derive a PWM to represent the LuxR binding specificity by using the highest-scoring 8-mer as a seed, as has been described previously (Berger *et al*., 2006); however, we found the motif too long to be adequately initialized by eight informative positions. Therefore, we used more conventional motif finding approaches to identify sequence motifs that were overrepresented among the brightest probes on each microarray. We used MultiFinder (Huber and Bulyk, 2006) to identify motifs specific to the 50 brightest probes on each array design. We generated a single LuxR motif by pooling the 50 brightest probes from each array (100 sequences total) and choosing the motif exhibiting the most significant group specificity score (Hughes *et al*., 2000).

### EMSA

The 500 bp double-stranded DNA probes were amplified from *V. harveyi* genomic DNA with 5′fluorescein-labelled primers, and the probes were gel-purified using the Zymoclean gel DNA recovery kit (Zymo Research). Each 20 μl binding reaction contained 10 nM probe, 50 ng μl^−1^ poly dIdC (Sigma), 10 mM HEPES (pH = 7.5), 100 mM KCl, 2 mM DTT and 200 μM EDTA. LuxR was added to achieve final concentrations of 10, 50, 100 and 250 nM, and reactions were incubated at 30°C for 10 min before loading onto a 5% TAE-polyacrylamide gel. Gels were visualized on a Storm 860 imaging system (Molecular Dynamics).

### Generation of promoter mutants

pCMW342 (Waters and Bassler, 2006) contains 350 bp surrounding the *qrgB* promoter fused to *gfp*. pKT1046 (gift of Kim Tu) contains 275 bp upstream of the transcriptional start site of the *qrr*4 gene fused to *gfp*-LVA (Andersen *et al*., 1998), an unstable variant of GFP. Promoter mutations were engineered into these two plasmids using the Quikchange XLII Site-Directed Mutagenesis kit (Stratagene).

### Analysis of promoter fusions

pCMW342 (and corresponding mutant constructs) and predicted LuxR-controlled target gene promoter fusion constructs were introduced by conjugation into *E. coli* S17-1 λ*pir* carrying either pLAFR2 or *luxR* on pLAFR2 (pKM699). Fluorescence production from *qrgB* promoters was measured in overnight cultures on a BD FACSAria cell sorter. pKT1046 and corresponding mutant constructs were introduced by conjugation into *E. coli* KT1190 (*luxO* D47E) carrying either pLAFR2 or pKM699. Overnight cultures were back-diluted 1:100 and grown to OD_600_ ≈ 0.8 in order to measure fluorescence production from the *qrr*4 promoters in late log-phase.

### Fluorescence anisotropy

5'fluorescein-labelled DNA oligonucleotides and their unlabelled complements were obtained from Integrated DNA Technologies. Double-stranded DNA probes were annealed by heating equimolar amounts of complementary single-stranded oligonucleotides in annealing buffer (Integrated DNA Technologies) at 94°C for 2 min, after which the reaction mixtures were slowly cooled to room temperature. Each 100 μl binding reaction was prepared in triplicate and contained 10 nM double-stranded DNA probe and 20 ng μl^−1^ poly dIdC in 10 mM HEPES (pH = 7.5), 100 mM KCl, 2 mM DTT, 200 μM EDTA and 100 μg ml^−1^ BSA. LuxR was added to achieve final concentrations between 10 and 400 nM. Reactions were incubated at 30°C for 35 min. Samples were excited at 480 nm and emission measured at 535 nm on a Perkin Elmer EnVision plate reader at 30°C. Each plate was read three times at 5 min intervals. Millipolarization (mP) was calculated as mP = 1000 × (S − G × P)/(S + G × P) where S = emission parallel to the excitation filter, P = emission perpendicular to the excitation filter and G (grating) factor is specific to both the instrument and the assay and is used to correct for variation between the detectors in each plane. For the assays presented here, G factor = 0.98.

*K*_d_ values were calculated as the concentration of LuxR at the half-maximal fractional change in fluorescence anisotropy (Head *et al*., 1998; Levine *et al*., 2007). Curves were fit by non-linear regression. (*F* − *F*_0_)/*F*_0_ = *B*_max_ × [LuxR]/(*K*_d_ + [LuxR]), where *F* = mP at the given concentration of LuxR, *F*_0_ = mP in the absence of protein, *B*_max_ = the maximum fractional change in fluorescence anisotropy and *K*_d_ is the apparent dissociation constant.
