# Supplementary material for: The Vibrio harveyi master quorum-sensing regulator, LuxR, a TetR-type protein is both an activator and a repressor: DNA recognition and binding specificity at target promoters
Source: Mol Microbiol. 2008 Aug 15;70(1):76–88. doi: 10.1111/j.1365-2958.2008.06389.x (PMC2628434; doi:10.1111/j.1365-2958.2008.06389.x)
Supplement: Supplementary file 1 [file mmi0070-0076-SD1.pdf]

Supplemental Experimental Procedures

Analyzing the protein binding microarray (PBM) data

The PBM experiments yielded a fluorescence value for each spot on the array. The fifty sequences with highest fluorescence from each array design (100 sequences total) were collectively analyzed using MultiFinder (Huber & Bulyk, 2006). This program, which integrates four different previously developed motif discovery algorithms, can identify multiple position weight matrices (PWMs) and has the user-specified option (which we employed here) to output the single PWM with the most significant group specificity score (which here corresponds to the PWM that is most specific to the input sequences as compared to the rest of the sequences on the arrays). Within these 100 sequences, MultiFinder identified a 21 bp over-represented motif. The resulting PWM described the binding specificity by assigning a probability for each base at each of the 21 nucleotide positions.

To identify LuxR binding sites within known directly regulated promoters from *V. harveyi*, we used this PBM-derived PWM in conjunction with MotifLocator (Thijs *et al.*, 2001), which scans for potential binding sites. MotifLocator uses as inputs the PWM, a background model of the genome, a chosen threshold probability score, and a list of target sequences, and then outputs a list of the target sequences that are above the input threshold score. Using MotifLocator, we analyzed both the known LuxR-regulated promoters and the PBM sequences using a variety of thresholds. We found that high threshold probabilities yielded too few expected hits, while low thresholds resulted in high false positive rates. Modifying the PWM by reducing it to a 20 bp sequence and

enforcing symmetry between the visibly important 5 bp half-sites did not significantly improve overall performance. We concluded that a more sophisticated approach was needed to identify the true LuxR binding sites, and therefore applied a machine learning algorithm called a Support Vector Machine (SVM) (Bishop, 2006).

Refining the binding-site model

SVMs are a family of machine learning algorithms that map data sets into higher dimensions to separate the data points into classes. This form of supervised learning is well-studied and software implementations are publicly available. The first step in training a classification SVM model is to define positive and negative examples. In our case, these examples were obtained from the PBMs. Specifically, we used the PWM from MultiFinder with a low enough threshold probability (85% confidence) to return at least one 21 bp subsequence for each LuxR-bound 60-mer sequence on the array. We considered LuxR-bound sequences to be those whose normalized fluorescence was greater than 20,000 fluorescence units, closely corresponding to the top 50 sequences from each array. To be conservative, we considered as unbound sequences those whose fluorescence was below a cutoff of 7,500 fluorescence units. This cutoff was chosen to optimize the performance of the SVM on its own data, using leave-on-out validation. For each of the bound array sequences, the highest scoring 21 bp subsequence was taken as a positive example for the SVM. As negative examples, we used all above-threshold 21 bp subsequences in each unbound sequence from the array. Together, the two PBM experiments yielded 131 positive examples and 1135 negative examples after reverse complementation. These positive and negative examples were converted into 42 digit

binary sequences in which each set of 2 digits represented one base (A = 00, C = 01, G = 10, T = 11). During training, the SVM software mapped the data into higher dimensions using a pre-determined kernel function. In the biological context, these higher dimensions represented specific co-dependencies between different positions within the motif, which are not considered in the PWM. We combined two SVM algorithms and averaged their scores to make predictions. This combination included the publicly available SVM light program (Joachims, 1999) and NEC's proprietary MiLDe software (NEC Laboratories, Inc.). After mapping the data into higher dimensions, the SVM algorithm found the hyperplane that best separated the positive from the negative examples. Distance from the hyperplane was used as a score to predict whether new examples were positive or negative. In the SVM light program, we used a 4th-order polynomial kernel for mapping, which allows co-dependencies of up to 4 bases. The use of even higher-order polynomials did not improve separation of the positive and negative examples. MiLDe allowed use of a radial basis kernel, which allowed us to prioritize reducing the number of false positives. Both SVM light and MiLDe have internal performance metrics based on the leave-one-out principle, which trains the model with all data points except one, and then tests the model on the example that was left out. This process was repeated on every data point to determine the precision and recall of the model (see Results).

Scanning and scoring promoter sequences

The first step in scanning promoter sequences for putative LuxR-binding sites was the same as the first step of SVM training. We scanned the promoter sequences using the PWM with the same threshold (>85%) and identified the above-threshold 21 bp

subsequences, which were then converted into binary form. The binary sequences were scored by the trained SVMs and the average score for each 21 bp subsequence was compiled. Sequences with scores greater than 0 were considered true binding sites, while scores less than 0 indicated false positives. To perform the *in silico* mutagenesis of the *qrr*4 and *qrgB* LuxR-binding sites, we calculated the average SVM scores for the appropriately modified 21 bp sequences.

Identification of novel genomic targets

We scanned the *V. harveyi* genome for putative binding sites using our dual-layered (PWM/SVM) scoring system. Although we expect a large fraction of the sites with positive SVM scores to bind LuxR, we focused on sites located within 300 bp of a putative gene. The *V. harveyi* genome has been computationally annotated for open reading frames (ORFs) by the sequencing center at Washington University using the program GeneMarkHMM (Lukashin & Borodovsky, 1998). We used this information to compile a list of approximately 200 candidate LuxR binding sites, on both *V. harveyi* chromosomes. To enrich for functional sites, we evaluated conservation of the promoter regions, as well as the downstream ORFs themselves, with respect to *V. harveyi*'s closest sequenced relative, *V. parahaemolyticus*. By eliminating candidate binding sites for which the respective promoter regions were less than 80% conserved, we shortened the candidate gene list to approximately 40 genes. With input from an energetic model (Kinney *et al.*, 2007), we picked five of the highest-scoring of these 40 sequences to test for LuxR-dependent regulation.

Table S1:

Model	True positives	False positives
PWM (85%)	98.86%	2.495%
PWM (90%)	64.77%	0.068%
PWM (95%)	7.95%	0.002%
PWM (85%) / NEC SVM	98.88%	0.049%
PWM (85%) / SVM Light	96.45%	0.140%

True positives = % of true positives predicted to be positive
False positives = % of true negatives predicted to be positive

Table S2: Oligonucleotides used for fluorescence anisotropy

Oligonucleotide name	Sequence
Consensus	actga TATTGATAAATTTATCAATAA tgact
Negative control	actga CTGACTGACTGACTGACTGAC tgact
qrgB WT	tgttta TATTGAGTTCACAATCAATAC cgatca
qrgB A2C	tgttta TCTTGAGTTCACAATCAATAC cgatca
qrgB A6C	tgttta TATTGCGTTCACAATCAATAC cgatca
qrgB A17C	tgttta TATTGAGTTCACAATCCATAC cgatca
qrgB A2CA17C	tgttta TCTTGAGTTCACAATCCATAC cgatca
*qrr*4 WT	cattt TTCTGATAAATGTATTAGTAG caatg
*qrr*4 A6C	cattt TTCTGCTAAATGTATTAGTAG caatg
*qrr*4 T15C	cattt TTCTGATAAATGTACTAGTAG caatg
*qrr*4 A17C	cattt TTCTGATAAATGTATTCGTAG caatg
*qrr*4 A6CA17C	cattt TTCTGCTAAATGTATTCGTAG caatg
VP0057/8	aacat TACTGATAAATTAGATATTTA tggct
VP0944/5	tagag TTAGTATCAATTTAATCAATA agata
VPA0197/8	taagg TAAATAATTATTTTAACAATA attaa
VPA0226/7	accaa AATTGATAAAATGAATAATTA gatat
VPA0649	attcc TTATTTACCAATTTATAAACT atgaa

Supplemental References

Bishop, C. M., (2006) *Pattern recognition and machine learning*, p. 738 p. Springer, New York.

Huber, B. R. & M. L. Bulyk, (2006) Meta-analysis discovery of tissue-specific DNA sequence motifs from mammalian gene expression data. *BMC bioinformatics* **7**: 229.

Joachims, T., (1999) Making large-Scale SVM Learning Practical. In: Advances in Kernel Methods - Support Vector Learning. B. Schölkopf, C. Burges & A. Smola (eds). Cambridge, MA: MIT-Press, pp. 169-184.

Kinney, J. B., G. Tkacik & C. G. Callan, Jr., (2007) Precise physical models of protein-DNA interaction from high-throughput data. *Proceedings of the National Academy of Sciences of the United States of America* **104**: 501-506.

Lukashin, A. & M. Borodovsky, (1998) GeneMark.hmm: new solutions for gene finding. *NAR* **26**: 1107-1115.

Thijs, G., M. Lescot, K. Marchal, S. Rombauts, B. De Moor, P. Rouze & Y. Moreau, (2001) A higher-order background model improves the detection of promoter regulatory elements by Gibbs sampling. *Bioinformatics (Oxford, England)* **17**: 1113-1122.
